# GLP-1 Receptor Agonists in Addiction: From Observational Signals to Research Priorities

**DOI:** 10.1192/bjo.2026.11941

**Published:** 2026-06-30

**Authors:** Augusta Okoro

**Affiliations:** St Andrew’s Healthcare, Northampton, United Kingdom

## Abstract

**Aims:**

Recent large registry studies report associations between GLP-1 RA use and reduced substance-related harms. We synthesize this evidence, identify critical knowledge gaps, and define research priorities needed before clinical translation.

**Methods:**

A targeted evidence synthesis was conducted across three domains:

1. Large registry studies examining outcomes in people with AUD or opioid use disorder (OUD) prescribed GLP-1 RAs.

2. Real-world cohort studies comparing overdose and hospitalisation rates between GLP-1 RA users and non-users.

3. Neuroscience literature describing GLP-1 receptor activity in reward-related brain regions.

**Results:**

Multiple observational studies show associations between GLP-1 RA use and reduced substance-related harms.

In a Swedish cohort of 227,866 individuals with AUD, semaglutide was associated with reduced alcohol-related hospitalisations (Lähteenvuo 2024). US data (>500,000 OUD, >800,000 AUD patients) showed lower opioid overdose and alcohol intoxication rates among GLP-1 RA users (Qeadan 2024; Wang 2024).

All current human findings are observational, but several human randomised controlled trials are now underway, including trials of semaglutide and exenatide for AUD, semaglutide for cocaine use disorder, and liraglutide for nicotine dependence.

The overlap between metabolic and addiction pathways may explain these associations, as GLP-1 signaling modulates reward processing relevant to both food intake and substance use. Preclinical studies demonstrate GLP-1R-mediated reduction in substance self-administration via reward pathway modulation in VTA, NAcc, and PFC. While this provides biological plausibility, observational human data cannot establish causality, and RCT evidence is needed.

**Conclusion:**

Observational data suggest potential associations between GLP-1 RA use and reduced substance-related harms, but cannot establish causality due to confounding. Before clinical application, essential research includes: (1) completion of ongoing RCTs with addiction-specific outcomes, (2) safety and tolerability evaluation in actively substance-using populations, including assessment of nausea/vomiting risks and drug interactions with methadone, buprenorphine, and benzodiazepines, (3) adherence and implementation feasibility studies in populations with chaotic substance use, and (4) health economics modeling. Psychiatrists should monitor emerging trial results critically as this evidence base develops in the coming years.

